# US racial and sex-based disparities in firearm-related death trends from 1981–2020

**DOI:** 10.1371/journal.pone.0278304

**Published:** 2022-12-14

**Authors:** Lindsay J. Young, Henry Xiang

**Affiliations:** 1 University of Cincinnati College of Medicine, Cincinnati, Ohio, United States of America; 2 Center for Pediatric Trauma Research, Nationwide Children’s Hospital, Columbus, Ohio, United States of America; 3 Center for Injury Research and Policy, Nationwide Children’s Hospital, Columbus, Ohio, United States of America; 4 Department of Pediatrics, The Ohio State University College of Medicine, Columbus, Ohio, United States of America; Stony Brook University, Graduate Program in Public Health, UNITED STATES

## Abstract

**Background:**

Firearms cause the most suicides (60%) and homicides (36%) in the US. The high lethality and availability of firearms make them a particularly dangerous method of attempted violence. The aim of this study was to study US trends in firearm suicide and homicide mortality and years of potential life lost before age 75 (YPLL-75) between 1981 and 2020.

**Methods:**

Data in this cross-sectional study were collected between 1981 and 2020 from the Centers for Disease Control and Prevention (CDC)’s WISQARS database for fatal injury and violence. Data from the US population were considered for all age groups and were divided by racial groups and sex for analysis.

**Results:**

Those most heavily impacted by firearm homicide were Black, with homicide age-adjusted death rates almost seven times higher than White people. A spike in firearm homicide deaths occurred between 2019 and 2020, with Black people having the largest increase (39%). White people had the highest rates of firearm suicide, and suicide death rates increased between 2019 and 2020. Increases in homicide and suicide YPLL-75 between 2011 and 2020 had most heavily impacted minority populations. Men had a firearm suicide rate that was seven times higher than women, and a firearm homicide rate that was five times higher than women.

**Conclusion:**

This study demonstrated that Black and White men were most impacted by firearm deaths, and that firearm homicide and suicide rates increased between 2019 and 2020 for all racial groups except Asian/Pacific Islander. Our results suggest that prevention efforts should focus on specific demographic factors and articulate the urgency to mitigate firearm-related deaths in the US.

## Introduction

Firearm injuries and deaths are increasingly recognized as a unique threat to the US population. Public attention focuses on mass shootings and their devastating impact on communities and has brought conversations on gun violence and ownership to the forefront of public health. Mass shootings represent only a small proportion of life lost to gun violence, as firearm suicide represents 60% and firearm homicide 36% (where mass shootings make up 0.2% of all homicides) of all firearm-related deaths [1, 2]. The US has the highest rate of firearm ownership globally, as Americans own 46% of the entire global stock of civilian firearms but make up 4% of the global population [3]. Approximately 1/3 of American households have firearms in their home, and studies have shown a disconnect between perceived safety of firearm ownership and data linking increased firearm mortality to keeping a gun in the home [4–6]. US firearm ownership is on the rise, as gun sales increased by 64% in 2020 as compared to the previous year. The first quarter of 2021 reported an 18% increase in firearm sales compared to the first quarter of 2020 [7].

Increasing rates of US firearm deaths have been attributed to the rising number of firearm suicides [8]. Consistent with increasing suicide death rates, suicidal ideation and behaviors are also increasing [9]. Firearms are used in only 5% of suicide attempts but account for 50% of suicide deaths due to the lethality of the method as 90% of firearm suicide attempts result in death [10]. Overall suicides occur most frequently among White men and people 70 years or older [11, 12]. Overall, two firearm suicides occur for every one firearm homicide [13]. Of all homicide deaths in the US, 73% of them are caused by firearms, and victims of firearm homicide are mostly young, male, and Black [14, 15], with Black men having the highest injury fatality rate [11]. Life expectancy for Black men is 5 years lower than White men and has been attributed to high homicide rates [16]. Poulson, et al. suggested that the underlying factors for high rates of homicide among Black men have not been adequately studied and hypothesized that structural racism, such as redlining and poverty, is linked to increased firearm violence [17].

There are a limited number of studies that examine historic trends in US homicide and suicide rates [8, 18], as previous publications provided isolated snapshots in time or studied homicide or suicide alone [11, 13, 14, 19]. Other studies focused primarily on mortality rates and rarely considered other factors impacting population-level health such as years of potential life lost. We aimed to assess racial and sex-based disparities by analyzing US firearm-related homicide and suicide trends in age-adjusted deaths rates and years of potential life lost. Our study was unique in examining 40 years of data to reveal a comprehensive understanding of trends and disparities of firearm-related mortality and loss of potential life, as well as the impact of the COVID-19 pandemic on firearm injuries.

## Methods

### Study design

For this cross-sectional study, age-adjusted death rates and YPLL-75 were used to describe trends and disparities of firearm-related suicide and homicide deaths between 1981 and 2020 in the US.

### Data source

Data were collected from the CDC’s Web-based Injury and Statistics Query and Reporting System (WISQARS), utilizing Fatal Injury Reports and Years of Potential Life Lost (YPLL) [20]. Because WISQARS is a de-identified publicly available database, IRB approval was not needed for this study. Data were coded using the International Classification of Diseases, Ninth Revision (ICD-9) for the years 1981–1998 and ICD-10 for the years 1999–2020. Firearm-related homicide (excluding legal intervention) and suicide age-adjusted death rates were calculated using year 2000 as the standardized year, with subgroup categories accounting for race, gender, and year. YPLL before age 75 were calculated by considering the current average lifespan of US population. Thus, YPLL-75 was collected for each year and racial group for firearm-related suicide and homicide (excluding legal intervention) deaths.

### Data analysis

Data collected from WISQARS were compiled into Microsoft Excel tables to organize age-adjusted death rates for firearm suicide and homicide by race, sex, and year of death. We also obtained firearm-related suicide and homicide YPLL-75 and stratified the data by year. First, we presented firearm-related suicide and homicide age-adjusted death rate trends and YPLL-75 by race between 1981 and 2020. We calculated ratios between racial groups for firearm homicide and suicide age-adjusted death rates between 1981 and 2020. The data were divided into 4 periods (1981–1990, 1991–2000, 2001–2010, and 2011–2020). Next, we calculated percent change of firearm YPLL-75 between each time period by the total US population and racial groups. We calculated yearly number of YPLL-75 by racial groups and their standard deviations using data between 1981 and 2020. We then calculated ratios between racial groups for firearm-related homicide and suicide YPLL-75 between 1981 and 2020. Finally, we showed firearm-related suicide and homicide age-adjusted death rate trends by sex between 1981 and 2020. We calculated age-adjusted death rates between 1981 and 2020 and found ratios between sex-specific firearm homicide and suicide death rates.

## Results

**Fig 1:**
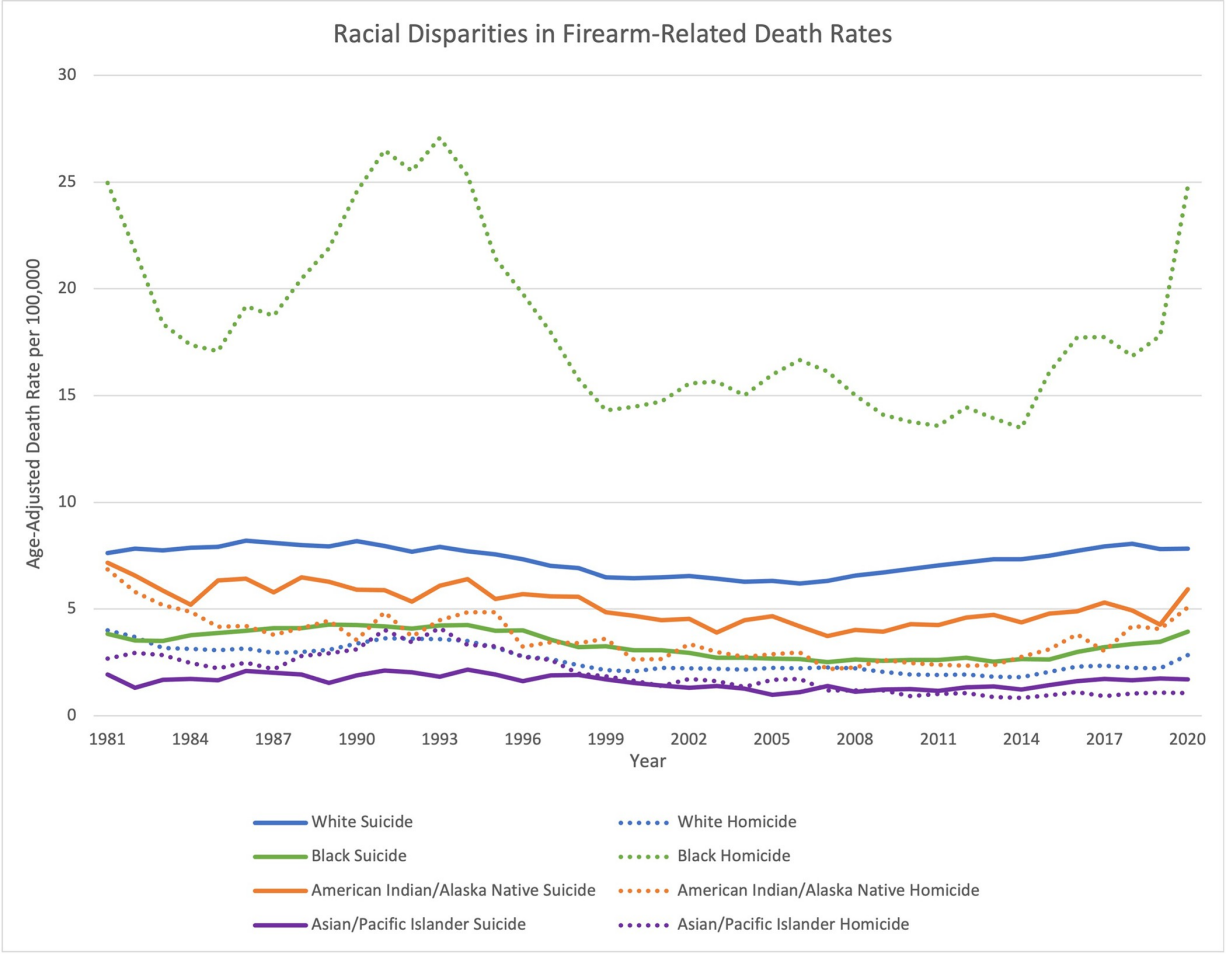
Yearly firearm-related age-adjusted death rates by race. Age-adjusted death rates per 100,000 for firearm-related deaths by racial groups from 1981 to 2020.

The highest yearly firearm-related death rate between 1981 and 2020 within a racial group was consistently homicide of the US Black population (**Fig 1**). The Black population had a firearm-related homicide death rate that was on average seven times higher than the White population. Rates of firearm-related homicide mortality within the Black population were five times higher than the next highest homicide death rate (American Indian/Alaska Native population). Among all populations, there was a rise and fall in firearm-related homicide death rates that occurred between 1990–1995. Between 2019 and 2020, a spike in firearm-related homicide death rates was seen in all racial groups except for Asian/Pacific Islander population (Black: 39%, White: 28%, American Indian/Alaska Native: 25%). Firearm-related suicide death rate was highest among the White population. The rate of firearm-related suicide mortality in the White population was two times higher than the Black population and was 1.4 times higher than the next highest firearm-related suicide death rate (American Indian/Alaska Native population). Between 2019 and 2020, firearm-related suicide death rates increased among all populations except Asian/Pacific Islander population and had the larger increases in the American Indian/Alaska Native population (39%) and Black population (14%).

**Fig 2:**
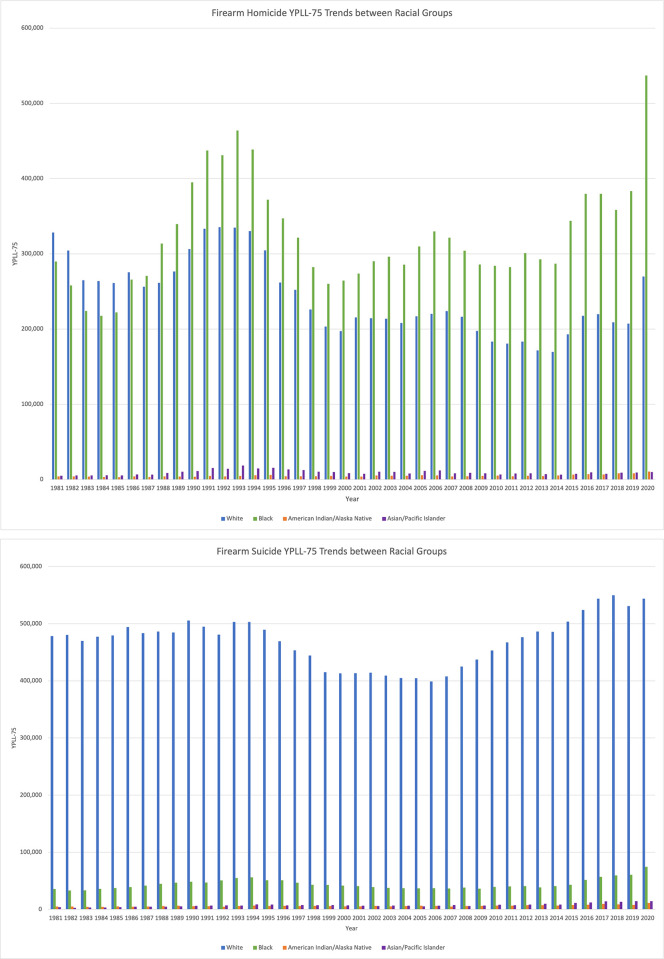
a) Firearm homicide YPLL-75 by race 1981 to 2020. b) Firearm suicide YPLL-75 by race 1981 to 2020.

Trends in firearm homicide YPLL-75 shown in **Fig 2A** followed a similar pattern of the firearm age-adjusted homicide death rate within the US Black population (**Fig 1**). A rise and peak were observed in the early 1990s followed by a decline, with a recent increase in firearm homicide YPLL-75 between 2019 and 2020. The firearm homicide YPLL-75 were higher among the Black population (mean = 323,471 ± 69,614) than the White population (mean = 242,699 ± 49,672), with a 1.3 times higher value. On average, firearm homicide YPLL-75 in the White population were twenty-six times higher than the next highest (Asian/Pacific Islander). **Table 1** shows firearm YPLL-75 and percent change between 4 time periods: 1981–1990, 1991–2000, 2001–2010, 2011–2020. Between 2011 and 2020, the largest increases in firearm homicide YPLL-75 occurred in the Black population (147%) and American Indian/Alaska Native population (90%). Overall, firearm suicide YPLL-75 had an upward trend since 2000 (**Fig 2B**). However, firearm suicide YPLL-75 had increased the most among minority populations (Asian/Pacific Islander: 97%, Black: 86%, American Indian/Alaska Native: 75%) as compared to the White population (16%), although White people made up the largest proportion of firearm suicide YPLL-75.

**Table 1:** Years of potential life lost under 75 (YPLL-75) and percent (%) change by firearm homicide and suicide deaths, 1981 to 2020 by 10-year interval periods.

Firearm Homicide	White	Black	American Indian/Alaska Native	Asian/Pacific Islander	Total
1981 YPLL-75	328,223	289,571	4,154	4,941	626,889
1990 YPLL-75	306,348	394,926	3,662	11,213	716,149
% Change	-6.66	36.38	-11.84	126.94	14.24
1991 YPLL-75	333,245	437,395	4849	15,238	790,727
2000 YPLL-75	197,211	264,359	3,888	8,514	473,972
% Change	-40.82	-39.56	-19.82	-44.13	-40.06
2001 YPLL-75	215,399	273,624	3,945	7,639	500,607
2010 YPLL-75	183,304	284,023	5,083	6,690	479,100
% Change	-14.90	3.80	28.85	-12.42	-4.30
2011 YPLL-75	180,737	282,407	4,300	8,004	475,448
2020 YPLL-75	269,847	536,966	10,622	9,875	827,310
% Change	49.30	90.14	147.02	23.38	74.01
**Firearm Suicide**					
1981 YPLL-75	478,321	35,758	5,044	3,790	522,913
1990 YPLL-75	505,514	48,301	5,711	6,041	565,567
% Change	5.69	35.08	13.22	59.39	8.16
1991 YPLL-75	494,600	47,027	5,517	6,742	553,886
2000 YPLL-75	412,916	41,540	5,253	7,122	466,831
% Change	-16.52	-11.67	-4.79	5.64	-15.72
2001 YPLL-75	413,229	40,832	5,260	6,474	465,795
2010 YPLL-75	452,915	39,435	6,814	8,080	507,244
% Change	9.60	-3.42	29.54	24.81	8.90
2011 YPLL-75	467,134	40,158	6,515	7,247	521,054
2020 YPLL-75	543,715	74,483	11,366	14,267	643,831
% Change	16.39	85.47	74.46	96.87	23.56

**Fig 3:**
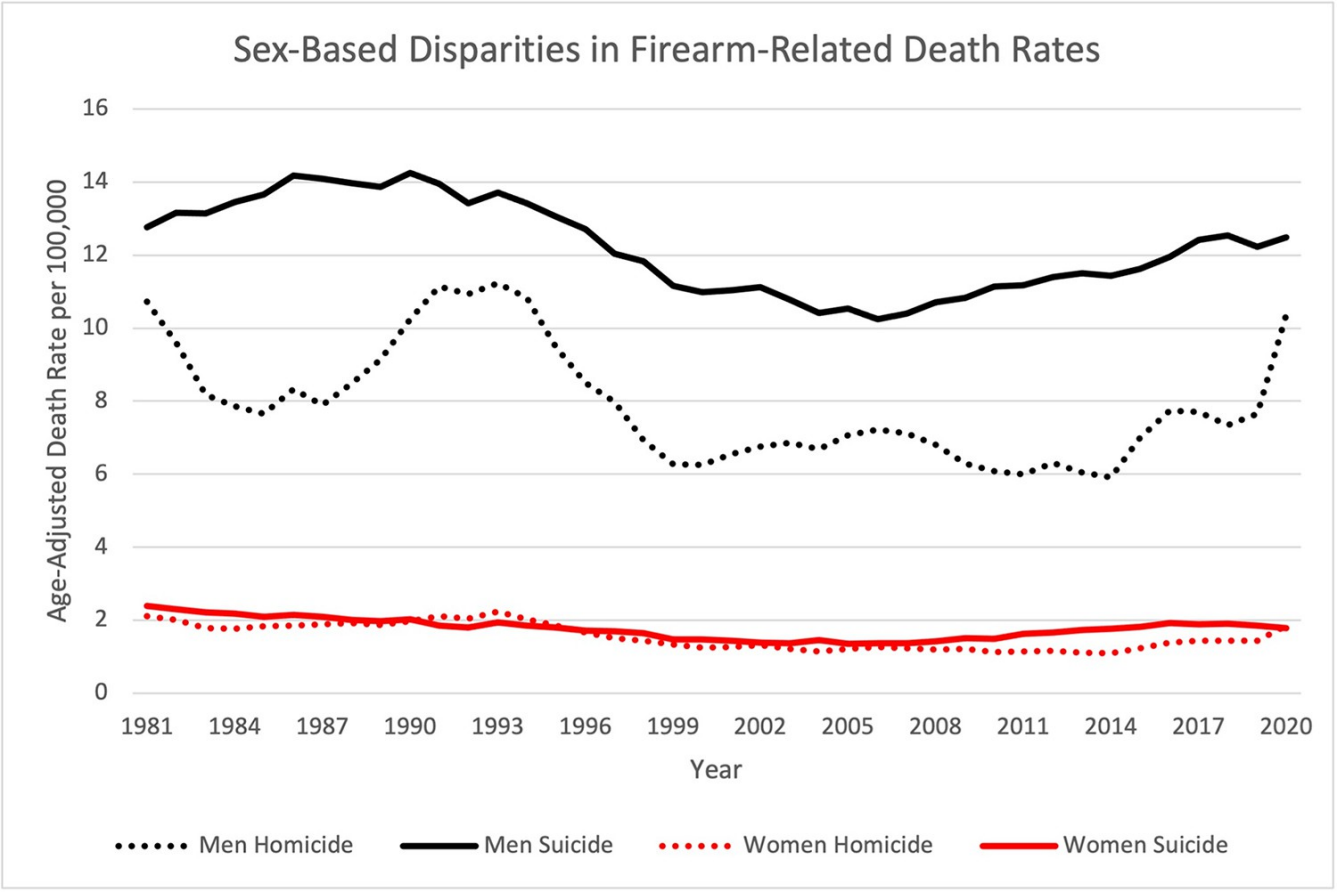
Yearly firearm-related age-adjusted death rates by sex. Age-adjusted death rates per 100,000 for firearm-related deaths by sex from 1981 to 2020.

The highest firearm-related death rate between sexes was seen in firearm suicide rate in men (**Fig 3**), and it closely resembled the White population’s firearm age-adjusted suicide death rate trend seen in **Fig 1**. The male firearm-related homicide rate was five times higher than the female rate. The male firearm suicide death rate was almost seven times higher than the female rate. Male firearm suicide death rate was 1.5 times higher than the male firearm homicide death rate. Female firearm suicide death rate was 1.1 times higher than the female firearm homicide death rate. Between 2019 and 2020, there was a 36% increase in male firearm homicide death rate and 29% increase in female firearm homicide death rate. During this same period, female firearm suicide death rate decreased by 3% and male firearm suicide death rate only increased by 2%.

## Discussion

Analyses of 40 years of data between 1981 and 2020 revealed that US firearm suicide and homicide deaths largely occurred among White men and Black men, respectively. The Black population was heavily affected by firearm homicide deaths. The differences in firearm homicide death rates were higher between racial groups as compared to firearm suicide deaths. From 2019–2020, there was a concerning spike in firearm homicide death rates across all racial groups, except for the Asian/Pacific Islander population, with the Black population experiencing a 39% increase in firearm homicide death rate during this spike. During the last decade, the American Indian/Alaska Native population showed increasing firearm homicide and suicide death rates and had the second highest rates for both firearm homicide and suicide deaths, despite being a small proportion (1.7%) of the US population [21]. Between 2019 and 2020, firearm homicide death rates increased at an alarming amount among both sexes, with males experiencing a 36% increase and females a 29% increase. However, firearm suicide death rate decreased in women and only increased marginally in men (2%) over this same time period.

Previous studies have examined racial and sex-based disparities in firearm-related deaths, but our study is unique in analyzing 40-years of deaths and YPLL-75 to elucidate the increasing firearm impact on the US population, which might not otherwise be reflected in studies using short periods of mortality data. Furthermore, analyzing 2019–2020 mortality data also allowed us to assess the impact of increasing violence and firearm ownership during the COVID-19 pandemic. Fowler, et al. found that men of any race and Black people were most likely to be the victims of firearm-related deaths [13]. Our results on sex disparities of firearm death rates are consistent with Fowler’s, where both studies found a male to female firearm suicide death ratio of 7:1, and male to female firearm homicide death ratio of 5:1. Fowler’s data were collected between 2010 and 2012; however, their results seemed to hold when expanded to our additional years of data [13]. A study by Sorenson stated that sex disparities are greater than racial disparities [18]. Conversely, our study found firearm homicide death rate to be nearly seven times higher in the Black population as compared to the White population, whereas the male to female firearm homicide death ratio was five times higher.

In a report of global data from 2016, homicides accounted for 64% and suicides 27% of firearm-related deaths [22]. There had been no change in all cause homicide death rates between 1990 and 2016, but firearm suicide death rates decreased globally at an annual rate of 1.6% [22]. However, in the US, firearm suicide death rates and YPLL-75 had shown an increasing trend between 2005 and 2018. In 2016, the US represented 35.3% of global firearm-related suicide deaths despite having only 4.3% of the global population [22]. Firearm-related deaths are a particular concern in the US as the firearm-related death rate is 11.4 times higher in the US than other high-income countries. Overall suicide rates between the US and other high-income countries are similar, but the US firearm suicide rate is almost ten times higher. The US overall homicide death rate is 7.5 times higher than other high-income countries, driven by a firearm- homicide death rate that is twenty-five times higher [14]. Considering the recent spike in firearm-related homicide death rate, it is important to understand what has been the driving force behind the particularly high rates of firearm deaths during the COVID-19 pandemic.

Changes in age-adjusted death rates reflect the need to focus on minority (particularly Black and American Indian/Alaska Native) populations’ vulnerability to firearm-related violence. Results from our study suggested that firearm homicide was a leading cause of death within the US population and is of particular concern given a spike in firearm death rates between 2019 and 2020. Men are heavily affected by firearm-related violence, and it is important for prevention efforts to target the demographics of people most affected by firearm-related homicide and suicide, respectively. Understanding how policies impact death rates could help develop legislations to decrease US firearm-related deaths. Sen and Panjamapirom conducted a study to understand the relationship between background checks for gun purchases and rates of firearm deaths and reported that firearm homicide deaths were lower in states checking for restraining orders and fugitive status, whereas firearm suicide deaths were lower in states checking for mental status, fugitive status, and misdemeanors [23]. Studies have shown that increasing availability of mental health care, reducing firearms in homes, and using secure storage have potential to reduce firearm-related deaths [24, 25].

### Limitations

This study had several limitations. First, we examined historical trends in firearm-related mortality rates and YPLL-75 and therefore causation could not be established. Second, we did not consider US Hispanic versus non-Hispanic populations as there were no Hispanic origin data available on WISQARS prior to 1990. Third, WISQARS provided only 4 racial groups (White, Black, American Indian/Alaska Native, and Asian/Pacific Islander) and no multiracial considerations when choosing “race” as an output group in fatal injury data, leaving out other racial groups that might have been impacted by firearm-related homicide and suicide deaths.

## Conclusions

This study found a recent spike in firearm homicide death rates between 2019 and 2020, particularly affecting men and the Black population. A national priority in dismantling structural racism is important to address disparities in firearm violence as indicated by our study.
